# The Relationships of Fibrinogen and C-Reactive Protein With Gait Performance: A 20-Year Longitudinal Study

**DOI:** 10.3389/fnagi.2022.761948

**Published:** 2022-04-12

**Authors:** Zohar Heumann, Iaroslav Youssim, Rachel Kizony, Yechiel Friedlander, Tamar Shochat, Ram Weiss, Hagit Hochner, Maayan Agmon

**Affiliations:** ^1^School of Nursing, Faculty of Social Welfare and Health Sciences, University of Haifa, Haifa, Israel; ^2^Braun School of Public Health, Hebrew University of Jerusalem, Jerusalem, Israel; ^3^Department of Occupational Therapy, Faculty of Social Welfare and Health Sciences, University of Haifa, Haifa, Israel; ^4^Department of Occupational Therapy, Sheba Medical Center-Tel Hashomer, Ramat Gan, Israel; ^5^Department of Pediatrics, Ruth Children’s Hospital, Rambam Medical Center, Haifa, Israel

**Keywords:** dual-task, cognition, gait speed, inflammaging, inflammation, C-reactive protein, fibrinogen, executive functions

## Abstract

**Background:**

Gait speed, a central marker of aging, has been linked to various health outcomes, such as cognitive and physical functions in middle-aged adults. Although long-term systemic low-grade inflammation is considered a mechanism underlying a variety of aging-related risk factors, the longitudinal associations between inflammation markers and gait speed are yet to be fully investigated.

**Objective:**

To explore the associations of CRP and fibrinogen levels, measured two decades ago, with gait speed among community dwelling adults, considering the contribution of cardio-metabolic factors and cognition.

**Methods:**

Study participants took part in two phases of the of the “Kibbutzim Family Study” (i.e., Phase II, 1999–2000 and Phase III, 2017–2019). Blood samples collected in Phase II (baseline) were used to determine level of inflammatory markers. Gait speed was assessed under single-task (ST) and dual-task (DT) conditions in Phase III. Demographic, anthropometric and clinical data were collected in both phases. Linear regression models were used to assess the adjusted associations of inflammation and gait speed.

**Results:**

A total of 373 individuals aged 34–99 (mean 64 ± 13 years) in Phase III were included in the study. Gait speed under ST was negatively associated with baseline levels of fibrinogen (*b* per standard deviation (SD) = −0.053, *p* = 0.0007) and CRP (*b* per SD = −0.043, *p* = 0.010), after adjusting for baseline and concurrent cardiometabolic risk factors. Accounting for executive functions, associations of fibrinogen with gait under ST were somewhat attenuated, yet associations remained statistically significant (*p* < 0.05). Associations with CRP were attenuated to the null. In contrast, there were no associations between inflammation markers and gait under DT.

**Conclusion:**

Our findings demonstrate that in a sample including younger to older adults, higher systemic inflammatory activity was linked with gait 20 years later, beyond age and cardiometabolic health, and to a certain extent, beyond executive functions. Thus, systemic inflammation may serve as an early marker to identify individuals at risk for gait decline.

## Introduction

Decreased mobility poses significant challenges for older adults and is a major public health problem for the rapidly growing aging population (Verghese et al., 2011; Prince et al., 2016; Tangestani Fard and Stough, 2019). Decline in gait performance, a central aspect of mobility is associated with reduced health status, decreased muscle strength, limited functional abilities (Potter et al., 1995; Fritz and Lusardi, 2009; Studenski et al., 2011), falls (Studenski et al., 2011; Ambrose et al., 2013), and cognitive decline (Peel et al., 2019). Although traditionally gait was considered as an automatic, in recent years it is clear that gait and cognition are intertwined (Montero-Odasso et al., 2019). Indeed, control of gait is achieved by a delicate equilibrium between automatic response and executive control. Limited cognitive capacity can affect gait performance, especially when the environment or the task is more challenged and attention resources are required (Clark, 2015). This link is pronounced throughout the aging process (Bridenbaugh and Kressig, 2011; Ambrose et al., 2013).

A plethora of factors are associated with age-related gait abnormalities. They include deterioration in muscle mass, aerobic capacity, insulin resistance (Volpato et al., 2012), cognition (Atkinson et al., 2007), executive function (especially attention) (Yogev-Seligmann et al., 2008), sensory and perceptual function, and mental health (Fritz and Lusardi, 2009). Long-term systemic low grade inflammation was proposed as a potential mechanism underling aging associated decline (Yaffe et al., 2003; Said, 2006; Schram et al., 2007; Oddy et al., 2018; Furman et al., 2019; Lasselin et al., 2020). This mechanism is also termed “inflammaging” (Franceschi and Campisi, 2014; Kennedy et al., 2014). Inflammaging is promoted by social, psychological, environmental and biological factors (Furman et al., 2019), such as a decline in levels of sex hormones after menopause or cumulative oxidative damage (Singh and Newman, 2011).

Inflammation is characterized by a systemic and local release of cytokines and chemokines that influence various cell types, like interleukin (IL)-6, which induces the expression of several hepatocyte genes that encode serum proteins, such as the inflammation markers fibrinogen and C-reactive protein (CRP). It is unclear whether they actually contribute to the causal pathway leading to disease rather than being markers for ongoing inflammation (Hager et al., 1994; Singh and Newman, 2011).

The role of inflammation markers has been investigated in relation to cognitive (Schram et al., 2007; Marioni et al., 2009; Beydoun et al., 2018; Tangestani Fard and Stough, 2019) and physical functions (Pedersen, 2019). With respect to cognition, a previous review reported cross-sectional associations between higher levels of inflammation markers and cognitive decline (Singh and Newman, 2011). However, findings from longitudinal studies are somewhat conflicting, with some reporting negative associations between inflammation markers and cognitive function (Marioni et al., 2009; Singh and Newman, 2011), but others reporting either none (Singh and Newman, 2011) or limited associations (Schram et al., 2007). As for physical function, the association with inflammation among older adults has been explored (Cesari et al., 2004; Roenneberg et al., 2004; Verghese et al., 2011; Kuh et al., 2019; Kositsawat et al., 2020). While most of these studies describe a negative association between inflammation markers and physical function, due to a large heterogeneity between studies (i.e., various inflammation markers and diverse measures of physical performance) generalizability is limited. Furthermore, only a few attempts have been made to address the specific link between inflammation and gait deterioration during the aging process. A rare example is a study in older adults (aged 70–79 at baseline) demonstrating that inflammation, measured by IL-6, is associated with slower gait in a cross-sectional and in a 10-year longitudinal analysis (Brown et al., 2016). Studies in younger and middle-aged populations are even more scarce, with one notable example of a study in middle-aged women, which found negative associations between CRP (in longitudinal and cross-sectional analysis) and fibrinogen (in cross-sectional analysis) and gait performance, measured as percent time spent in double support, resulting in slower gait speed (Tomey et al., 2009).

Importantly, to the best of our knowledge, no study has yet explored the impact of inflammation on simultaneous mobility and cognition. Simultaneous impact on multiple domains can be studied using the Dual-Task paradigm (Clark, 2015). For instance, gait speed can be measured while walking as an ST, and together with a simultaneous cognitive task as DT. When two tasks are conducted simultaneously, they compete for the same brain resources (Muir-Hunter and Wittwer, 2016), and therefore the DT condition often yields poorer performance than the ST condition in either or both domains (Clark, 2015). As most daily motor tasks are performed simultaneously with another task, such as walking while talking or carrying a cup of coffee, assessment of gait within the DT paradigm may enhance the ecological validity of gait evaluation (Bridenbaugh and Kressig, 2011). Reduced gait speed during ST and DT situations is a reliable, valid, sensitive, and specific predictor of survival among older adults (Potter et al., 1995; Fritz and Lusardi, 2009; Studenski et al., 2011) and can predict Alzheimer’s (Verghese et al., 2011), Parkinson’s disease (Belghali et al., 2017) and an accelerated aging process even at the age of 45 (Rasmussen et al., 2019). In sum, further investigation of the relationship of inflammation markers with mobility and cognition in a middle-aged and older adult is warranted. Understanding the contribution of inflammation markers to gait deterioration throughout the aging process may promote better health outcomes for the aging population.

The current population-based longitudinal study aims to determine the associations of CRP and fibrinogen levels, measured two decades ago, with gait performance under ST and DT conditions measured in adults currently aged 34–99, accounting for baseline and current cardiometabolic health measures as well as for executive functions.

## Materials and Methods

### Data

The Kibbutzim Family Study (KFS) was established to investigate the environmental and genetic basis of cardiometabolic risk factors, described previously (Friedlander et al., 2006; Granot-Hershkovitz et al., 2018). At the time of recruitment, the participants belonged to large families living in 6 Northern Israeli kibbutzim, which are close-knit communal settlements. Kibbutzim have created a relatively homogeneous environment for their members. For example, earnings were uniformly distributed, Kibbutzim members typically dined jointly and healthcare was regularly and continuously provided to all members *via* clinics located within each Kibbutz. Kibbutzim members are mostly of Ashkenazi Jewish ancestry, with the remaining members belonging to other Jewish subgroups. Thus, the KFS is a useful resource for the study of independent associations between risk factors and health outcomes, above and beyond the contribution of shared socioeconomic and behavioral factors.

This work focuses specifically on data collected at two time points: (1) 1999–2000 (Phase II, or referred to as baseline in the current analysis, *n* = 922), and (2) the Kibbutzim Aging (KAG) study which took place in 2017–2019 (Phase III). Of the 922 members who participated in the baseline examination (Phase II) 127 had died and 246 had left the Kibbutz. The remaining members were invited to participate in the current study and 373 individuals were then assessed (response rate 68%). These individuals belong to 111 original families (3.4 members per family on average).

All participants provided informed consent, and the study was approved by the Institutional Review Board of the Hadassah-Hebrew University Medical Center in Jerusalem and of the Faculty of Social Welfare and Health Sciences, University of Haifa.

### Tools

#### Gait Speed Under Single-Task and Dual-Task

Gait performance was assessed in phase III (KAG study) as Single-Task (ST) walking, a 1-min walk on a ten-meter straight and flat surface, and as Dual-Task (DT) walking, a 1-min walk on the same surface with an added cognitive load (i.e., consecutive subtraction by 7 from a random 3-digit number). Gait parameters were evaluated using three small wireless OPAL movement sensors (Mobility Lab, APDM Inc., Portland, Oregon) affixed to the participant’s legs and waist; these sensores have been shown to be sensitive and reliable (Schmitz-Hübsch et al., 2016). Gait speed (meters/second) was measured separately for ST and DT and correct cognitive responses were recorded for both tasks. The tasks were administered in a random order and the participants were instructed to conduct both tasks at their best performance, i.e., to complete as many passes as they can (while walking and not running) and to compute (out load) correctly as many numbers as they can. No instructions for prioritization were given.

#### Inflammation Markers

Fibrinogen and CRP concentrations were measured in Phase II (baseline) from blood samples collected after a 12-h overnight fast from an antecubital vein into a 3.8% sodium citrate-containing tube, which was stored at −80°C until assayed. The study measurements were performed under quality control supervision of established reference laboratories, and 5% blind duplicate samples were used to estimate the analytic variation within runs and over time (Friedlander et al., 2006).

#### Covariates

In both phases information on age, sex, and diagnosis of hypertension, hyperlipidemia and diabetes based on use of corresponding medications was collected from participants using a self-administered questionnaire. Standing height and weight were measured at the two time points.

Executive functions were evaluated with two assessments: (a) the Trail Making Test–B (TMT–B) that measures complex visual scanning and cognitive flexibility (Reitan, 1985; Atkinson and Therneau, 2007) and (b) the Color-Word Stroop Test (CWST) measures selective attention and response inhibition. In the current study, we calculated part 3, the interference trial, since it required the higher level of selective attention and response inhibition (Jensen and Rohwer, 1966).

### Statistical Analyses

Linear regression models were used to assess the association between levels of inflammatory markers measured in Phase II of the KFS (baseline) and gait speed (meter/second) assessed in Phase III (KAG study). The exposure variables were CRP and fibrinogen measurements analyzed as continuous variables as were the outcome variables, gait speed under ST and DT conditions. Gait variables were normally distributed.

Analyses were performed in three steps as follows. In the first step (model 1), analyses of inflammation markers and gait outcomes were adjusted for current age, sex and current height. In the second step (model 2), analyses were further adjusted for body mass index (BMI) measured at baseline (phase II) as well as for medication-treated hyperlipidemia, hypertension or diabetes at baseline. Finally, in the third step (model 3) the analyses were also adjusted for BMI and hyperlipidemia, hypertension or diabetes at the 20-year follow-up (phase III). These covariates were selected as they are known to be associated with CRP, fibrinogen, cognitive performance or gait speed (Ridker et al., 2001; Ravona-Springer et al., 2012; Mahinrad et al., 2020). Additionally, in the fully adjusted linear regression models we examined the associations of inflammation markers with gait speed under ST independent of executive functions. Regression coefficients and standard errors were scaled by standard deviation of corresponding exposure variables. All statistical analyses presented were performed using Stata 12 (Stata Corporation, College Station, TX, United States) and a *p*-value < 0.05 was considered statistically significant.

As aforementioned, the KFS was designed as a family based study and although sample attrition reduced the degree of relatedness in phase III compared with phase II, some study participants belonged to the same family and thus shared a genetic and environmental background potentially resulting in higher degree of similarity in phenotypes between family members (Cannon et al., 2001; McCoach, 2010; McCoach and Adelson, 2010; Bae et al., 2014). To test whether we would need to account for relatedness in this sample, we compared results using ordinary linear regression models, which assume independent observations, to those obtained using linear mixed-effects kinship models, which control for relatedness (Almasy and Blangero, 1998; Visscher et al., 2008; Jamsen et al., 2013; Bae et al., 2014). Specifically, for the kinship models, we used *lmekin* in the R package coxme, which implements a linear mixed-effects kinship model fitted by Residual Maximum Likelihood using the generalized Cholesky decomposition of the relatedness matrix (Atkinson and Therneau, 2007; Jamsen et al., 2013). We found that the two modeling approaches yielded identical results and therefore, reported findings are based on ordinary linear regression models. We also examined the functional form of the relationships between the exposure variables (i.e., inflammation) and the outcomes using non-linear terms and quantiles of the exposure variables, but did not detect evidence for non-linearities.

## Results

Table 1 shows the characteristics of 373 participants (51% female) aged 34–99 (mean 64 ± 13 years) who took part in both phase II and III of the study. During 20 years of follow-up, the proportion of individuals treated for hyperlipidemia, hypertension or diabetes grew dramatically and mean BMI has also increased from 26.5 to 28.0 kg/m^2^. As expected, mean gait speed under ST was higher than that under DT (1.4 ± 0.3 and 1.2 ± 0.3, respectively).

**TABLE 1:** Sample characteristics.

Characteristics	Mean or%	SD	Minimum	Maximum
Female (%)	50.9			
**Measured in phase II (1999–2000)**				
Age, years	45.3	12.6	14.9	80.0
C-reactive protein, CRP (mg/L)	2.7	3.1	0.1	19.8
Fibrinogen (mg/DL)	277.3	74.5	90.0	519.0
BMI (kg/m^2^)	26.5	4.3	17.6	44.6
Medication-treated hyperlipidemia (%)	3.8			
Medication-treated hypertension (%)	8.0			
Medication-treated diabetes (%)	2.1			
**Measured in phase III (2017–2019)**				
Age, years	63.8	12.7	34.0	99.0
Height (cm)	164.9	9.4	136.0	189.9
BMI (kg/m^2^)	28.0	4.8	18.0	45.0
Medication-treated hyperlipidemia (%)	25.6			
Medication-treated hypertension (%)	26.6			
Medication-treated diabetes (%)	10.7			
Gait speed under ST, (m/s)	1.4	0.3	0.1	2.3
Gait speed under DT (m/s)	1.2	0.3	0.1	2.0
TMT–B (part 2 minus part 1), sec	52.3	38.7	−36.0	277.0
CWST (part 3), sec	39.4	10.8	10.0	86.0

*SD, standard deviation; ST, single task condition; DT, dual task condition; TMT–B, trail making test–B; CWST, color-word stroop test.*

Table 2 presents the results for linear regression models examining the association between baseline inflammation markers and gait performance 20 years later. Gait speed under ST was negatively and significantly associated with baseline levels of fibrinogen in all models. Specifically, in the fully adjusted model (Model 3), a standard deviation (SD) unit increase in baseline fibrinogen was associated with 0.053 m/s lower ST gait speed (*p* = 0.0007). Additionally, each SD unit increase in baseline CRP was associated with 0.043 m/s (*p* = 0.01) lower ST gait speed, after adjusting for all covariates. Variability in gait under ST explained by the fully adjusted models was substantial (*R*^2^ = 0.418 and 0.408 for fibrinogen and CRP, respectively). In contrast, while higher baseline levels of both inflammation markers were associated with lower DT gait speed, these effects were not statistically significant.

**TABLE 2:** Associations between inflammation markers and gait speed under ST and DT conditions 20 years later.

	Model	Fibrinogen b (95% CI)*	*p*	CRP b (95% CI)*	*p*
**Dependent variables**					
Gait speed ST, m/s	1	−0.061 (−0.091, −0.031)	**<0.001**	−0.048 (−0.079, −0.018)	**0.002**
	2	−0.051 (−0.081, −0.020)	**0.001**	−0.035 (−0.067, −0.002)	**0.036**
	3	−0.053 (−0.084,−0.023)	**0.0007**	−0.043 (−0.075,−0.010)	**0.010**

Gait speed DT, m/s	1	−0.024 (−0.054, 0.007)	0.129	−0.018 (−0.048, 0.012)	0.247
	2	−0.015 (−0.045, 0.016)	0.341	−0.003 (−0.035, 0.029)	0.859
	3	−0.024 (−0.055,0.007)	0.132	−0.018 (−0.051,0.014)	0.264

*CI, confidence interval.*

*Model 1 adjusted for age, sex, and current height.*

*Model 2 further adjusted for covariates at baseline: BMI, hypertension, hyperlipidemia and diabetes.*

*Model 3 further adjusted for covariates at 20-year follow-up: BMI, hypertension, hyperlipidemia and diabetes.*

*Coefficients and CIs scaled by standard deviation of the exposure variables.

*Significant findings are in bold.

To assess whether the associations between inflammation markers and gait under ST are independent of executive functions, we further introduced into the linear regression models the participants’ performance in two separate cognitive tasks, i.e., TMT–B and CWST (Table 3). Results show that after accounting for executive functions in either one of the tests, associations of fibrinogen with gait under ST were somewhat attenuated, yet remained statistically significant (*p* < 0.05), suggesting that the association of fibrinogen on gait speed is independent of executive functions. The associations with CRP, however, were attenuated to the null. Notably, no significant interaction was found between age, i.e., above and below 65 years old and neither CRP nor Fibrinogen with gait speed under ST and DT.

**TABLE 3:** Associations between inflammation markers at baseline and gait speed under ST, with further adjustment for executive functions at 20-year follow-up.

Model	Fibrinogen b (95% CI)*	*p*	CRP b (95% CI)*	*p*
**Gait speed ST, m/s**				
Model 1: adjusted for TMT–B (part 2 minus part 1)	−0.030 (−0.059, −0.001)	**0.041**	−0.019 (−0.050, 0.011)	0.211
Model 2: adjusted for CWST (part 3)	−0.030 (−0.059, −0.0005)	**0.047**	−0.021 (−0.052, 0.010)	0.177

*CI, confidence interval; TMT–B, trail making test–B; CWST, color-word stroop test.*

*Both models are adjusted for age, sex, current height and BMI, hypertension, hyperlipidemia and diabetes at baseline and at 20-year follow-up. Model 1 is further adjusted for executive function measured by TMT–B and Model 2 is further adjusted for executive function measured by CWST.*

*Coefficients and CIs scaled by standard deviation of the exposure variables.

*Significant findings are in bold.

## Discussion

This study was set to explore the longitudinal relationship of inflammation markers with gait under both ST and DT paradigms, considering the contribution of baseline and concurrent cardiometabolic health measures as well as executive functions. The results demonstrate that fibrinogen and CRP levels at baseline are significantly and negatively associated with gait speed, measured 20 years later, under ST conditions among adults, after controlling for age and past and present cardiometabolic health measures. Moreover, our findings show that after accounting for executive functions, fibrinogen, but not CRP, remained significantly associated with gait speed under ST conditions.

Overall, the longitudinal associations between inflammation markers and gait speed, especially when cognition is considered, have rarely been investigated. Among the few studies that explored this link, findings are mixed. For example, our findings are in line with a study that demonstrated negative associations of CRP and fibrinogen with gait speed in middle-aged women under ST conditions over a 5-year period (Tomey et al., 2009), and with other longitudinal studies, with follow-up of two (Verghese et al., 2011) and 6 years (Kositsawat et al., 2013) that found that IL-6 was negatively related to physical function (Verghese et al., 2011; Kositsawat et al., 2013, 2020; Brown et al., 2016). However, in a study of 977 participants aged 65 and above at baseline, no association was observed between CRP levels and physical function measured after three and 6 years (Kositsawat et al., 2020).

Interestingly, the findings of our study may point to an independent relationship between inflammation and gait, adjusted for cardiometabolic factors. Whereas the associations between non-communicable diseases, including cardiometabolic risk factors, and inflammation markers are well-established (Zeyda and Stulnig, 2009; Camps and García-Heredia, 2014; Park et al., 2014), the link between gait and cardiometabolic risk factors is inconsistently supported. For example, BMI is consistently and negatively associated with gait speed in both cross-sectional and longitudinal studies, while studies of associations of hypertension with gait speed report conflicting results (Figgins et al., 2021). Similarly, although it is well-documented that advanced diabetes alters gait (Allet et al., 2009; Holtzer et al., 2018), other studies provide mixed results (Figgins et al., 2021). This discrepancy can stem from sample heterogeneity between studies or varying degrees of diabetic complications, such as neuropathic changes and reduced vascularity. Nevertheless, in the current study, cardiometabolic factors measured at two time points over 20 years, only partially explained the link between inflammation and gait speed.

The link between higher inflammation markers and reduced gait performance, demonstrated in the current study, can be explained by an interplay between two pathways: the “sickness behavior” and the “brain aging.” The “sickness behavior” mechanism proposes that inflammation causes metabolic and neuroendocrine changes aiming to conserve metabolic energy and allocate more nutrients to the activated immune system (Furman et al., 2019; Lasselin et al., 2020). These changes lead to behaviors such as reduced food intake, altered sleep, increased blood pressure, insulin resistance, and dyslipidemia, which are crucial as part of a normal acute inflammatory response. However, in the case of low-grade non-infective systemic chronic inflammation, these changes can cause a breakdown of immune tolerance and collateral damage to tissues and organs over time, in part by inducing oxidative stress (Furman et al., 2019). These changes also have a catabolic effect on muscles, possibly leading to reduced grip power, and induce musculoskeletal disorders including osteoporosis, arthritis (Verghese et al., 2011), and sarcopenia (Furman et al., 2019). The “brain aging” mechanism suggests that inflammatory biomarkers affect brain functioning, which might influence both cognitive and motor performance (Verghese et al., 2011), as well as the interplay between them. Many studies have explored the comorbidity of motor and cognitive decline in older age, termed the Motor Cognitive Risk (MCR) syndrome (van Oijen et al., 2005), which is associated with higher risk for dementia (Montero-Odasso et al., 2019).

Another factor that can explain the link between inflammation and gait speed is cognition. In the current study, the effect of cognition was evaluated in two ways: by implementing the DT paradigm and by adjusting for executive functions during gait as ST. Contrary to our hypotheses, when a cognitive task was added (DT condition) both inflammation markers were not significantly associated with gait speed. In most studies using the DT paradigm to assess associations between gait speed and other aging-related factors, gait speed under the DT paradigm demonstrated either equivalent or stronger associations than during ST. For example, in a meta-analysis, associations of gait speed under both conditions with falls were equivalent among older adults (Menant et al., 2014). In a large sample of middle-aged adults, gait speed was associated with brain health and physical performance when evaluated under both conditions (ST and DT), yet the association with gait speed under DT was even stronger than ST (Rasmussen et al., 2019). There are a few possible reasons for the discrepancy observed between our findings for DT and that of previous studies. Typically, gait under the DT paradigm is analyzed as a marker for various aging-related outcomes such as falls (Muhaidat et al., 2013) and physical function (Schaefer and Schumacher, 2011), while in the current study the inflammation was considered a potential early marker for gait under DT. Another potential explanation may be related to differences in the cognitive tasks that were administrated (i.e., reciting the alternate letter vs. subtraction by seven). Most recently, a systematic review demonstrated that differences in gait under DT condition are more pronounced among older adults with cognitive decline than among the general population. Thus, the lack of association with DT seen in our study could be potentially explained by the fact that participants were not selected based on their health status or age and were relatively young when gait was assessed (mean age 62.3 years) and largely independent.

In the past, gait was considered to be exclusively under automatic spinal control, however, to date it is widely recognized that even simple walking requires cognitive resources, i.e., executive function (Clark, 2015). Thus, in this work, we also considered the contribution of executive function, i.e., attention, as an underlying mechanism in the associations of fibrinogen and CRP with gait during ST walking. The link between fibrinogen and gait speed remained significant after taking into account executive functions, whereas the association with CRP was diminished. These findings point to potentially different effects of fibrinogen and CRP on the motor and the cognitive aspects that are required for gait performance during the aging process, i.e., the mechanism underlying the link between CRP and gait speed can be explained by executive functions, while fibrinogen may also have an independent relationship with gait speed. Fibrinogen and CRP are acute-phase proteins that are used as non-specific markers for inflammatory disease (van Oijen et al., 2005), and both were shown to be associated with cognitive decline in previous studies (Schram et al., 2007; Marioni et al., 2009; Noble et al., 2010). However, fibrinogen also has an important hemostatic role affecting platelet aggregation and endothelial function (van Oijen et al., 2005), that in turn affect cardiometabolic functions which are associated with reduced physical capacity (Tay et al., 2019) and decreased muscle strength that relate to slower gait speed (Figgins et al., 2021).

This study has several limitations. The first is the homogenous sample of participants residing in a community with highly similar socioeconomic characteristics at baseline and some of which share family origin. The likelihood for bias due to family relatedness is low as it was controlled for in our statistical analyses, but generalization may nevertheless be limited due to similarities in ethnicity and socioeconomic status. Moreover, as this study was established to investigate cardiometabolic risk factors, and gait measurements were not well developed back then, it was not assessed at baseline, so as cognitive function. In fact, long-term associations of cumulative blood pressure with gait and cognitive function in midlife were recently examined in a well-established population-based cohort of cardiovascular disease in the United States (ref circulation). In that study too, baseline measurements of gait and cognition were not accounted for, as very often happens in population-based studies when examining associations spanning over decades (Mahinrad et al., 2020). Relatedly, the inflammatory markers assessed in this study are those most extensively investigated in relation to cardiovascular risk (Rosenson and Koenig, 2003), however, they are not necessarily the sole predictors of gait or cognitive changes associated with inflammaging. Future studies should explore these longitudinal relationships in other communities and in a large age-range and consider a broader set of inflammation markers to determine those most relevant to these changes over time. Lastly, although our study has adequate power to detect effects in the magnitude seen for gait under ST (85% power for fibrinogen and 70% for CRP), for effects of smaller magnitude, such as those observed for gait under DT, we may be underpowered and larger samples may be required.

To conclude, our findings demonstrate that higher systemic inflammatory activity is associated with gait 20 years later, beyond age and cardiometabolic risk factors and, to a certain extent, even beyond executive functions. Notably, this link was shown also in younger adults, and therefore suggests that systemic low-grade inflammation may serve as an early marker to identify individuals at risk for developing gait decline, resulting in accelerated aging and disability. Understanding the influence of inflammation on gait may help improve early identification, intervention, and prevention of mobility and cognitive decline, and consequently promote successful aging.

## Data Availability Statement

The raw data supporting the conclusions of this article will be made available by the authors, contingent on ethical approvals.

## Ethics Statement

The studies involving human participants were reviewed and approved by the Review Board of the Hadassah-Hebrew University Medical Center in Jerusalem and of the Faculty of Social Welfare and Health Sciences, University of Haifa. The patients/participants provided their written informed consent to participate in this study.

## Author Contributions

RK, YF, TS, RW, HH, and MA were responsible for the conception and design of this study. HH and MA supervised the project. ZH and IY headed data collection and conducted the analyses, guided by YF, HH, and MA. ZH, HH, and MA drafted the manuscript. IY, RK, YF, TS, and RW critically reviewed the manuscript. All authors contributed to the article and approved the submitted version.

## Conflict of Interest

The authors declare that the research was conducted in the absence of any commercial or financial relationships that could be construed as a potential conflict of interest.

## Publisher’s Note

All claims expressed in this article are solely those of the authors and do not necessarily represent those of their affiliated organizations, or those of the publisher, the editors and the reviewers. Any product that may be evaluated in this article, or claim that may be made by its manufacturer, is not guaranteed or endorsed by the publisher.
